# Supplementary material for: Discovery and application of insertion-deletion (INDEL) polymorphisms for QTL mapping of early life-history traits in Atlantic salmon
Source: BMC Genomics. 2010 Mar 8;11:156. doi: 10.1186/1471-2164-11-156 (PMC2838853; doi:10.1186/1471-2164-11-156)
Supplement: Additional file 2 — Information on developed 76 locus single-run INDEL panel in Atlantic salmon. Information on fluorescence labeling, primer concentrations, PCR pooling and links to alignments, INDEL motifs and GENESCAN (Burge and Karlin 1997) predictions of genes/exons are available in html format. [file 1471-2164-11-156-S2.ZIP › Additionalfile2/snpsummary4237.html]

```
Cluster 973 Contig 2

prev  Summary    Contig List  next
```

Size of Consensus sequence = 3101

Number of sequences = 24

Minimum redundancy = 6

Key

A gi|89861218|gb|DY717341.1|DY717341 EST\_ssal\_rgb2\_73080 ssalrgb2 mixed\_tissue Salmo salar cDNA Salmo salar cDNA clone ssal\_rgb2\_618\_131\_fwd 3', mRNA sequence  
B gi|24389370|gb|CA059127.1|CA059127 ssalrgb518116 mixed\_tissue Salmo salar cDNA, mRNA sequence  
C gi|84975191|gb|DW473592.1|DW473592 SGP315568 Atlantic salmon Spleen cDNA library Salmo salar cDNA clone MI5-3766 5', mRNA sequence  
D gi|89862427|gb|DY718550.1|DY718550 EST\_ssal\_rgb2\_74289 ssalrgb2 mixed\_tissue Salmo salar cDNA Salmo salar cDNA clone ssal\_rgb2\_622\_088\_fwd 3', mRNA sequence  
E gi|24341679|gb|CA040753.1|CA040753 ssalshc502123 spleen Salmo salar cDNA, mRNA sequence  
F gi|89861872|gb|DY717995.1|DY717995 EST\_ssal\_rgb2\_73734 ssalrgb2 mixed\_tissue Salmo salar cDNA Salmo salar cDNA clone ssal\_rgb2\_619\_319\_fwd 3', mRNA sequence  
G gi|85038064|gb|DW566242.1|DW566242 EST\_ssal\_rgb2\_30661 rgb2 Salmo salar cDNA clone ssal\_rgb2\_549\_271\_fwd 3', mRNA sequence  
H gi|85050180|gb|DW578358.1|DW578358 EST\_ssal\_rgb2\_42777 rgb2 Salmo salar cDNA clone ssal\_rgb2\_569\_131\_fwd 3', mRNA sequence  
I gi|89872004|gb|DY728127.1|DY728127 EST\_ssal\_rgb2\_83866 ssalrgb2 mixed\_tissue Salmo salar cDNA Salmo salar cDNA clone ssal\_rgb2\_637\_186\_fwd 3', mRNA sequence  
J gi|85052149|gb|DW580327.1|DW580327 EST\_ssal\_rgb2\_44746 rgb2 Salmo salar cDNA clone ssal\_rgb2\_572\_182\_fwd 3', mRNA sequence  
K gi|24386173|gb|CA055930.1|CA055930 ssalrgb519184 mixed\_tissue Salmo salar cDNA, mRNA sequence  
L gi|84986114|gb|DW536464.1|DW536464 EST\_ssal\_rgb2\_883 rgb2 Salmo salar cDNA clone ssal\_rgb2\_502\_212\_fwd 3', mRNA sequence  
M gi|24390367|gb|CA060124.1|CA060124 ssalrgb513048 mixed\_tissue Salmo salar cDNA, mRNA sequence  
N gi|89861219|gb|DY717342.1|DY717342 EST\_ssal\_rgb2\_73081 ssalrgb2 mixed\_tissue Salmo salar cDNA Salmo salar cDNA clone ssal\_rgb2\_618\_131\_rev 5', mRNA sequence  
O gi|84974869|gb|DW473270.1|DW473270 SGP314135 Atlantic salmon Spleen cDNA library Salmo salar cDNA clone MI5-3395 5', mRNA sequence  
P gi|85052148|gb|DW580326.1|DW580326 EST\_ssal\_rgb2\_44745 rgb2 Salmo salar cDNA clone ssal\_rgb2\_572\_182\_rev 5', mRNA sequence  
Q gi|85055175|gb|DW583353.1|DW583353 EST\_ssal\_rgb2\_47772 rgb2 Salmo salar cDNA clone ssal\_rgb2\_577\_049\_rev 5', mRNA sequence  
R gi|85038063|gb|DW566241.1|DW566241 EST\_ssal\_rgb2\_30660 rgb2 Salmo salar cDNA clone ssal\_rgb2\_549\_271\_rev 5', mRNA sequence  
S gi|57122304|gb|CX353745.1|CX353745 ssalrgb519184\_rev\_0 mixed\_tissue Salmo salar cDNA, mRNA sequence  
T gi|85050181|gb|DW578359.1|DW578359 EST\_ssal\_rgb2\_42778 rgb2 Salmo salar cDNA clone ssal\_rgb2\_569\_131\_rev 5', mRNA sequence  
U gi|84986113|gb|DW536463.1|DW536463 EST\_ssal\_rgb2\_882 rgb2 Salmo salar cDNA clone ssal\_rgb2\_502\_212\_rev 5', mRNA sequence  
V gi|89872003|gb|DY728126.1|DY728126 EST\_ssal\_rgb2\_83865 ssalrgb2 mixed\_tissue Salmo salar cDNA Salmo salar cDNA clone ssal\_rgb2\_637\_186\_rev 5', mRNA sequence  
W gi|29324691|gb|CB513465.1|CB513465 ssalrgb518116\_rev mixed\_tissue Salmo salar cDNA, mRNA sequence  
X gi|89862426|gb|DY718549.1|DY718549 EST\_ssal\_rgb2\_74288 ssalrgb2 mixed\_tissue Salmo salar cDNA Salmo salar cDNA clone ssal\_rgb2\_622\_088\_rev 5', mRNA sequence

4 SNPs detected

A B C D E F G H I J K L M N O P Q R S T U V W X  cosegregation weighted

474 . . T - T T T T - - T - - C T - . . . . . . . .   1/4 14.58
475 . . T - T T T T - - T - - T T - . . . . . . . .   3/4 43.75
476 . . C - C C C C - - C - - C C - . . . . . . . .   3/4 43.75
477 . . T - T T T T - - T - - T T - . . . . . . . .   3/4 43.75
